# Characteristics of Carbon and Kevlar Fibres, Their Composites and Structural Applications in Civil Engineering—A Review

**DOI:** 10.3390/polym16010127

**Published:** 2023-12-30

**Authors:** Ștefania Ursache, Camelia Cerbu, Anton Hadăr

**Affiliations:** 1 Department of Mechanical Engineering, Faculty of Mechanical Engineering, Transilvania University of Brasov, B-dul Eroilor No. 29, 500036 Brasov, Romania; stefania.olareanu@unitbv.ro; 2 Department of Strength of Materials, Faculty of Industrial Engineering and Robotics, National University of Science and Technology Politehnica Bucharest, 313 Splaiul Independentei, 060042 Bucharest, Romania; anton.hadar@upb.ro

**Keywords:** hybrid composite, carbon fibre, Kevlar fibre, beams strengthened, mechanical properties

## Abstract

Kevlar and carbon fibres and fabrics have won a leading place in the structure market, although such materials are not cheap, and are increasingly used for reinforcing and strengthening structural elements in the civil engineering, automotive, aerospace and military industries, due to their superior mechanical properties, especially in terms of strength. The mechanical characteristics of such composite materials must be known in order to numerically simulate the mechanical behaviour of such structures in terms of the distribution of stresses and strains. It has also become a necessity to understand the effects of reinforcement with both types of fibres (carbon fibres and Kevlar fibres) on the mechanical properties, especially on the impact properties of such composites. This review aims to expose the main advantages and disadvantages of the hybridization of carbon and Kevlar fibres. For this reason, an overview is presented concerning the main characteristics (tensile strength, flexural strength, impact strength, coefficient of thermal expansion and so on) for carbon and Kevlar fibres and also for hybrid Kevlar–carbon composite materials to aid in the design of such hybrid composite materials. Finally, some civil construction rehabilitation and consolidation applications of the composites reinforced with carbon fibre, Kevlar fibre or with hybrid Kevlar–carbon fabrics are highlighted in the last part of the paper.

## 1. Introduction

Nowadays, hybrid composite materials are used in many engineering applications due to their versatile properties such as low weight, strength to weight ratio, low cost, ease of structural development and high strength. For example, the automobile industry utilizes composites and hybrid composites in many interior and exterior applications [1].

The term “hybrid composite materials” refers to those composite materials which contain either different layers (each layer being reinforced with other types of fibres) or layers reinforced with hybrid fabrics made of two different types of fibres. Over the past few years, there has been continuous improvement in the performance of hybrid composite materials. Interest in hybridizing the materials in the composites into a common matrix has been growing since 2000 [2,3]. The evolution of hybrid composites has changed the way researchers look at composite materials, because those materials are capable of forecasting the mechanical properties that are similar to the properties of other conventional materials and sometimes more than that [4]. The research that has been carried out has discovered that the behaviour of hybrid composite materials seems to simply be the sum of the individual components’ properties, which are controlled by factors such as the nature of the matrix, fibre length, chemical composition of the reinforcements, fibre–matrix interface and hybrid design. In other words, through the careful selection of the reinforcement fibres and manufacturing technology, it is possible to meet various performance standards and requirements of such composites, with economic benefits [2,3,4,5,6,7,8,9,10].

Composite materials reinforced with Kevlar–carbon hybrid fabrics are a good example of high-performance properties in terms of strength-to-weight and stiffness-to-weight ratios, making them more practical for load-bearing applications in all industries. These lightweight hybrid composite materials may be exposed to low-velocity impact loadings during manufacture, normal operation and so on [7,11].

Aramid fibres, which include Kevlar fibres, are synthetic and manufactured on an industrial level because they require high-level technology, and are characterized by their high modulus of elasticity. In this study, it is shown that certain types of Kevlar fibre have a tensile strength comparable to carbon fibres. Compared to glass fibre and carbon fibre, which are the two most common types of fibre used as reinforcement fibres for composite materials, Kevlar fibre has a modulus of elasticity higher than that of glass fibres, but lower than that corresponding to carbon fibre. It has also been shown that the density of Kevlar fibre is lower than the densities corresponding to glass fibres and carbon fibres. Characterized by high performance, composite materials reinforced with Kevlar fibre are used in applications in different fields (aerospace, automotive, defence industry, medical) where it is necessary to ensure lightweight materials with good impact performance and also in applications where high strength, high stiffness and fatigue strength are also important [12,13,14]. It is also necessary to mention the high sensitivity of Kevlar fibres to humidity and ultraviolet (UV) rays, which leads to a decrease in the mechanical characteristics [15,16].

The main characteristics of the carbon fibres are the following: high stiffness-to-weight ratio, high strength-to-weight ratio, good corrosion resistance, electricity conduction and high melting temperature. Due to their high performance, there are many applications of composite materials reinforced with carbon fibre in different industries (aerospace, automotive, civil engineering), for which the lightweight materials are required [17,18,19,20,21,22,23,24].

Both types of fibre are characterized by high mechanical performance, so combining carbon fibre and Kevlar fibre in the same reinforcing material would lead to hybrid composite materials, which can be used in applications where structures with reduced thickness, low weight and high strength are needed. Moreover, the hybridization of carbon fibre and Kevlar fibre combines the advantages of the two types of fibre, and simultaneously mitigates their less desirable disadvantages [25,26,27].

However, based upon recent studies, it was concluded that the hybridization of carbon fibre with Kevlar fibre improves the mechanical behaviour of such hybrid composite materials in impact loading and reduces the post-impact strength losses in comparison with the carbon/epoxy composites [28,29,30].

The literature review shows that similar review papers published, with a similar topic, usually focus on one of the following research areas: manufacturing properties and applications of the carbon fibres and their composites [31,32,33,34]; properties and applications of aramid fibres and their composites including Kevlar fibres [12,35]; mechanical properties of hybrid composites [36]; strengthening of timber structural members with carbon- or glass-reinforced polymers [37,38]; strengthening of concrete structures with fibre-reinforced polymer composites [39,40,41]. Considering the papers published in the literature in recent years, addressing the applications of composite materials for the strengthening of different structural elements in building engineering (concrete and wooden beams, ballistic protection panels), it can be observed that most of the research which has focused on such applications use carbon fibres or Kevlar fibres or Kevlar–carbon hybrid fabrics. In this context, compared to other similar reviews, this review paper presents the present state-of-the-art beginning with the properties of carbon fibres and Kevlar fibres, continuing with their hybrid fabric forms and properties of Kevlar–carbon composites and ending with a review of the research in the field of civil structural elements (wood and concrete beams) strengthened with such fibres and composite materials. In addition, the effects of the hybridization of the carbon and Kevlar fibres in different hybrid Kevlar–carbon composite structures is also discussed by comparing their mechanical properties with ones of similar composite structures reinforced either with carbon fibres or with Kevlar fibres.

Taking into account the large-scale application of carbon and Kevlar fibres in the manufacturing of structural elements in civil engineering, researchers interested in this field need to be familiar with the main properties, advantages and disadvantages of such types of fibres and also the advantages of Kevlar–carbon hybrid composites for the perspective of developing of new structural elements. For example, to safely reinforce the structural elements, researchers and readers must know how sensitive Kevlar fibres are to the effects of heating rates or water absorption.

This paper presents a review on the main characteristics (mechanical properties in tensile, bending and impact tests; thermal properties; water absorption) of carbon fibres, Kevlar fibres, and also of Kevlar–carbon hybrid composites, in order to provide the references required for the research areas connected with such materials. The main purpose is to show the potential of carbon composites, Kevlar composites and Kevlar–carbon hybrid composite materials in order to use them for structural applications in civil engineering, based on their advantages concerning their high strength, stiffness and impact properties. For this purpose, this review intends to provide a summary of the main properties of the carbon and Kevlar fibres and of their composite materials, which make them recommendable for improvements in the strength and stiffness of structural elements.

The study of the state-of-the-art in the field of structural applications as strengthening elements for beams in civil engineering intends to provide the scientific background required for a future perspective regarding the development of new structural elements.

## 2. Properties of Kevlar Fibres

### 2.1. Mechanical Properties of Kevlar Fibres

After a review of the literature in the field of Kevlar, in Table 1, the tensile properties of the most common types of Kevlar fibres are summarized, which can be found on the market.

Analysing the data shown in Table 1, it can be noted that the density and the tensile strength vary within narrow ranges of values for all analysed Kevlar fibres while there are significant differences between the values of Young’s modulus.

### 2.2. Thermal Properties of Kevlar Fibres

In their paper, Li et al. [45] analysed the thermal degradation of Kevlar fibres in air and nitrogen by high-resolution thermo-gravimetric (TG) analysis. The technique, based on the principle that the maximum weight loss rate is determined for the minimum heating rate, provided thermal degradation results which were in excellent agreement with the values determined by traditional TG experiments. Kunugi et al. [46], from the experiments performed in their study, also concluded that the thermal degradation of Kevlar fibres begins at about 450 °C. In Table 2, the reported values in the literature are shown for the characteristics regarding the thermal degradation of Kevlar fibres.

Kevlar fibres subjected to high temperatures demonstrate a different behaviour in the axial and transverse directions, showing a very small negative coefficient of thermal expansion (CTE) in the longitudinal direction of the fibres and a positive CTE in the transverse plane. Rojstaczer et al. [48] have also demonstrated that the values obtained for CTE of Kevlar49 fibres are positive in the transverse direction and negative in the axial direction. These values of CTE are provided in Table 3 along with the values of CTE from the one other reference [16].

### 2.3. Impact Properties of Kevlar Fibres

Kevlar fibres are known as materials with very good impact strength. Kevlar fibres absorb a large amount of strain energy on impact and, as a result, are used for the manufacturing of ballistic protection panels and military protective equipment (bullet-proof vests, helmets, tactical gloves).

Friction between the filaments is considered one of the most important parameters for characterizing the ballistic performance of Kevlar fabrics. Friction between yarns (yarn-to-yarn friction) is generally evaluated by yarn pull-out force and is associated with impact strength. In their study, Dixit et al. [49] showed that the impact energy absorption of Kevlar fabric is approximately 103 J while the yarn pull-out force is about 20 N.

### 2.4. Influence of Water Absorption on Kevlar Fibres

Table 4 shows the effects of water absorption on both Young’s modulus and the tensile strength for three types of Kevlar fibres (Kevlar29, Kevlar49 and Kevlar149).

It can be seen that water absorption leads to a decrease of 14.5–37.6% in tensile strength compared to the value obtained for specimens tested before immersion in water. After saturation, the Young’s modulus is 8.6% smaller than the one obtained for the dried specimen for Kevlar29, while the water absorption at saturation was the highest. On the other hand, for Kevlar49 and Kevlar149 fibres, it is found that after saturation with water, the Young’s modulus is 48.3% and 29.9% lower than the value recorded for the dry specimens.

For Kevlar29 fibres, the decrease in both the fibre strength and Young’s modulus is smaller, although these absorbed more water.

### 2.5. Effects of Ultraviolet (UV) Light on Kevlar Fibres

It is well known from the literature [16,42,50], that, like other polymeric materials, Kevlar fibres are sensitive to UV light. Upon exposure, the yellow or gold Kevlar fibres turn firstly orange and then brown because of their degradation. The degradation of Kevlar fibres occurs only in the presence of oxygen and is not affected by other atmospheric contaminants. In his research, Mustafa [50] showed that if the Kevlar fabric is thicker, it is more protected, as the outer fibres form a protective barrier covering the inner fibres in a filament or fabric bundle. Another solution for UV protection is the dense packing of the fibre itself, with or without a matrix.

Considering that the UV stability of Kevlar fibres increases with their size (i.e., with the number of deniers for a yarn, with the thickness of the fabric or the diameter of a rope), Table 5 summarizes the data regarding the degradation of tensile strength for different sizes of Kevlar fibres, related to the exposure time to UV light.

### 2.6. Commercial Forms for Kevlar Fibres and Products

**Figure 1:**
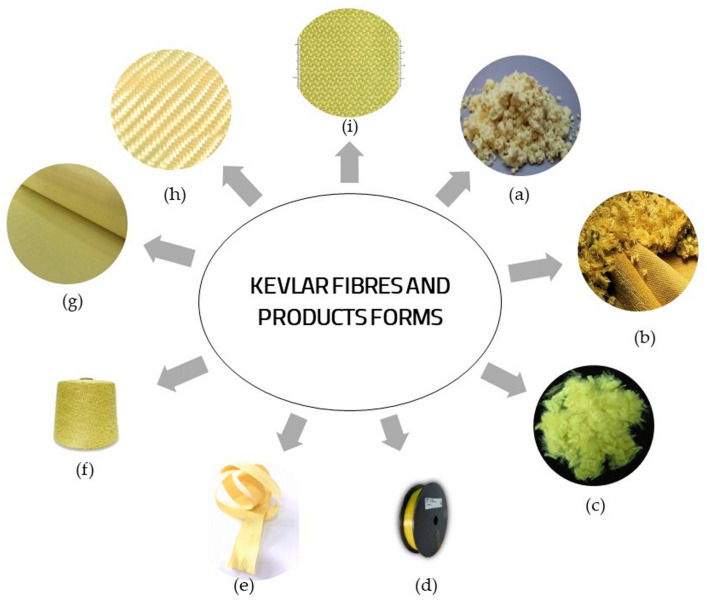
Kevlar fibre and product forms: (**a**) flock fibres; (**b**) short fibres; (**c**) pulp fibres; (**d**) spun yarn; (**e**) braided tape; (**f**) filament fibres; (**g**) plain fabric; (**h**) twill fabric and (**i**) satin fabric.

There are numerous commercial forms of products made of Kevlar fibres available in the market of building materials, as illustrated in Figure 1. The main types of Kevlar products include: short fibres or flock, continuous filament yarns, roving, woven fabrics.

In Figure 1a–c, three types of Kevlar short fibres (a brand for aramid fibres) are represented. These fibres are mainly used to reinforce different composite materials, typically dedicated for parts subjected to friction because it has been proven that the addition of aramid fibres leads to an improvement in the friction coefficient [51]. Kevlar short fibres are also recommended for ballistic applications such as armour made of high-density polyethylene (HDPE) reinforced with Kevlar short fibres [52]. It was shown that the additional reinforcement of the composite materials with Kevlar short fibres based on HDPE reinforced with wood flour leads to a significant increase in impact strength [53].

Table 6 summarizes the main properties of Kevlar short fibres.

In Figure 1d–f, some products made of continuous filaments of Kevlar fibres are shown.

Kevlar spun yarns can be either knitted or woven into various products. Continuous filaments of Kevlar fibres are the primary source for protective clothing in the ballistics industry and these can be woven into plain fabric (Figure 1g), twill fabric (Figure 1h) and satin weave fabric (Figure 1i).

Plain weave fabric (Figure 1g) is the most common textile which can be found on the market. In the plain weave fabric, each warp thread alternately passes over and under each weft thread.

In a twill weave fabric (Figure 1h), one or more warp threads are alternately woven, over and under two or more weft fibres in a regular and repeated sequence, forming a pattern of distinct diagonal lines. The main advantage of twill weave fabric is that, due to reduced wrinkling, the cloth has a flat surface and better mechanical properties than plain fabric.

A Kevlar satin weave (Figure 1i) is a typical type of fabric, with a glossy top surface and a dull back. It is not as durable as either plain or twill weave fabric.

## 3. Properties of Carbon Fibres

### 3.1. Mechanical Properties of Carbon Fibres

Compared with other synthetic fibres (glass, aramid, nylon fibres), carbon fibres have numerous advantages, including high stiffness, high tensile strength, light weight, high chemical resistance, high temperature stability and a low thermal expansion coefficient. Due to these properties, carbon fibres are widely used in the aerospace industry, civil engineering and for military and sports equipment. However, they are relatively expensive compared to other fibres such as glass fibres or plastic fibres. Carbon fibres exhibit exceptional properties such as high stiffness and specific strength, making them excellent reinforcements for composite materials [55].

Table 7 presents a classification of carbon fibres according to their mechanical properties.

Following the experimental measures carried out on different types of carbon fibres, it was concluded that the values of the transverse contraction coefficient (Poisson’s ratio) are in the range of 0.26–0.28 [61].

### 3.2. Thermal Properties of Carbon Fibres

Carbon fibres combine high tensile strength and high modulus of elasticity with low weight. Furthermore, this high tensile strength is maintained up to extremely high temperatures [62]. A decrease of 21.94% of the tensile strength (decreasing from 5.47 GPa down to 4.27 GPa) was recorded by heating at temperatures above 400 °C [63]. Simultaneously, above this temperature, a reduction in the fibre diameter of approximately 37% was recorded [64].

Carbon fibres withstand very high temperatures and their decomposition starts at a temperature above 400 °C and ends at a temperature of approximately 850 °C. Table 8 shows the influence of temperature on the tensile strength of carbon fibres extracted with examples from the literature. It can be seen that the carbon fibres denoted with ultra-high modulus (UHM) in Table 8 are relatively stable when heated at temperatures in the range of 350–450 °C. On the other hand, it is reported that the tensile strength decreases by 43.5% on heating at a very high temperature (Table 8).

Carbon fibres subjected to high temperatures exhibit a different behaviour in the axial and transverse directions, showing a very small coefficient of thermal expansion in the longitudinal direction of the fibres and a positive coefficient in the transverse plane [32,56,66,67].

Table 9 [66] summarizes the values of these coefficients for six different types of carbon fibres. It can be seen that negative values of the coefficient of thermal expansion were reported for carbon fibres in the longitudinal direction (Table 9). Conversely, the coefficients of thermal expansion in the transverse direction of the carbon fibres were positive and higher than those measured in the longitudinal direction (Table 9).

### 3.3. Electrical Conductivity of Carbon Fibres

Carbon fibres are good electrical conductors and have a much longer service life than the metal cables. The capability of carbon fibres for transmitting electrical power is closely related to the graphitization process at different temperatures [18]. As the graphitization temperature increases, the electrical conductivity increases. Table 10 shows the values of electrical conductivity according to the graphitization temperatures [68].

### 3.4. Carbon Fibres and Product Forms

**Figure 2:**
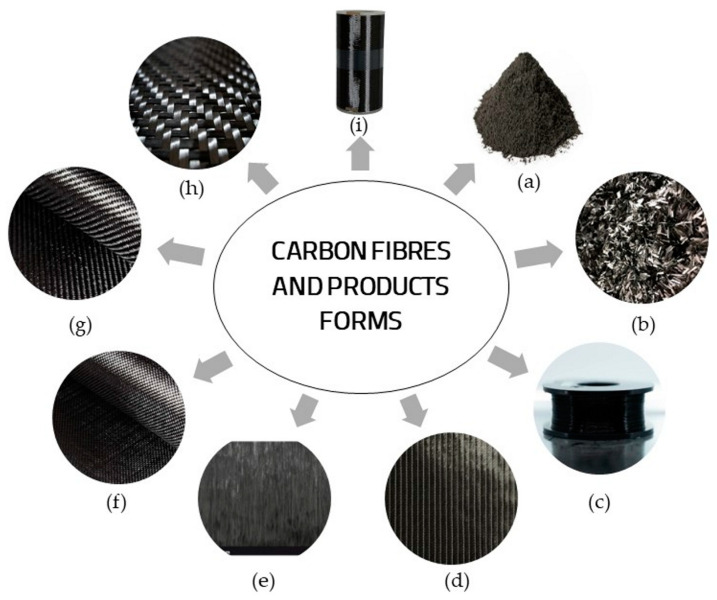
Carbon fibre and product forms: (**a**) milled fibres; (**b**) chopped fibres; (**c**) filament fibres; (**d**) biaxial fibres; (**e**) unidirectional fibres; (**f**) plain fabric; (**g**) twill fabric; (**h**) satin fabric and (**i**) unidirectional cloth.

There are numerous commercial forms for carbon fibres and products, as shown in Figure 2.

Milled carbon fibres (Figure 2a) are short shapes of carbon fibres, typically about 100 µm in length, with improved properties in terms of the electrical conductivity.

Short and chopped carbon fibres (Figure 2b) have the advantages of high tensile strength, abrasion resistance, corrosion resistance, good conductivity and high temperature resistance. Carbon fibre filaments (Figure 2c) have gained popularity due to their reliability, heat resistance and stiffness. These are used worldwide for the 3D printing of parts and tools across various industries. In Figure 2(d) and Figure 2(e), the most common product types of carbon unidirectional fibres used in the manufacturing carbon-fibre-reinforced plastics (CFRPs) are presented. These CFRPs find applications in various industries [69], and are often used as laminates with titanium alloy in the aircraft industry (for fuselages and wings) [70].

Plain weave carbon fabrics (Figure 2f) are recommended for manufacturing flat sheets, tubes and three-dimensional curve surfaces, due to their high fabric stability.

Twill weave carbon fabrics (Figure 2g) consist of 2 × 2 or 4 × 4 patterns and are the most common and widely sold fabrics due to the fact that these fabrics are pliable and can form complex surfaces while maintaining a good stability. For example, Hadar et al. [22] used twill 2 × 2 carbon fabric to reinforce epoxy vinyl ester resin of type 470–300 Derakane Momentum to investigate the mechanical properties of the single ply and multi-ply CFRP subjected to tensile and bending tests.

Composite materials reinforced with carbon satin fabrics (Figure 2h) are used to form complex surfaces.

## 4. Kevlar–Carbon Hybrid Fabrics

### 4.1. Plain Weave Hybrid Kevlar–Carbon Fabrics

**Figure 3:**
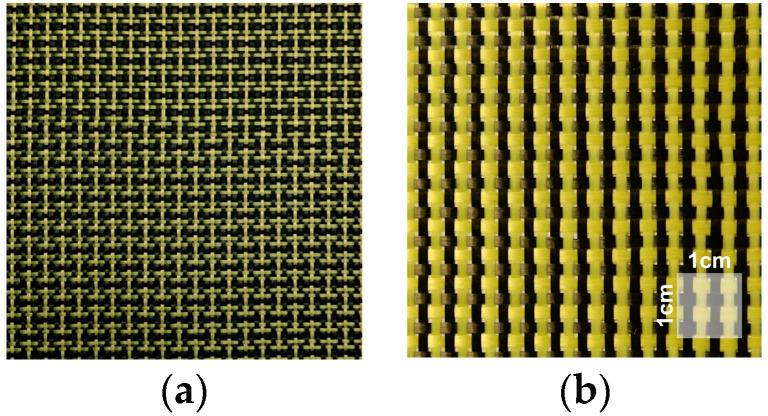
Plain hybrid Kevlar–carbon fabrics: (**a**) plain hybrid Kevlar–carbon fabric with a density of 188 g/m^2^ [71]; (**b**) plain hybrid Kevlar–carbon fabric with a density of 163 g/m^2^ [72].

The plain hybrid Kevlar–carbon fabric (Figure 3) combines the properties of the two types of fibres, offering high levels of strength and stiffness, as well as high impact strength. This type of fabric can be easily used for reinforcing composite materials in applications, which require high mechanical performance, such as in the construction of canoes and panels for rally cars.

### 4.2. Twill Weave Hybrid Kevlar-Carbon Fabrics

**Figure 4:**
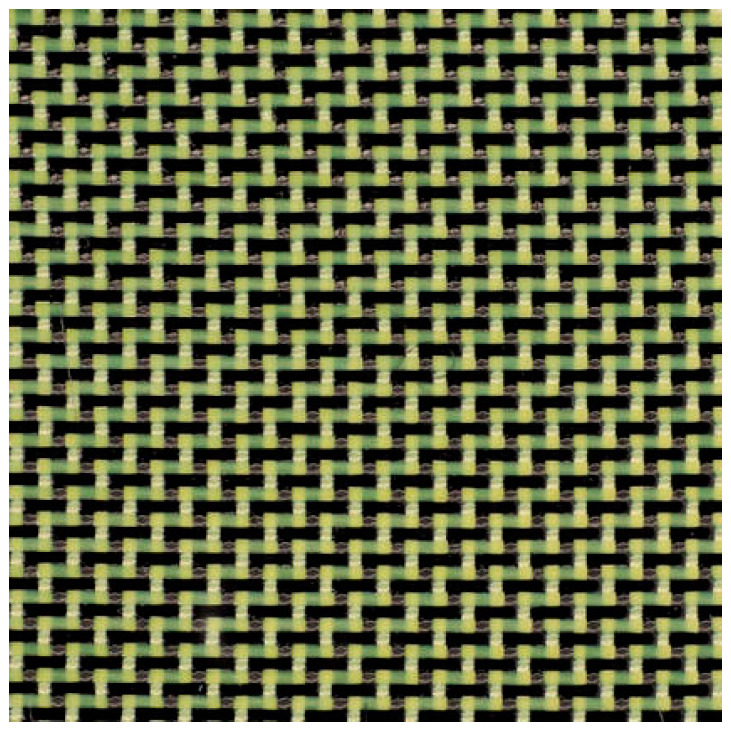
Twill hybrid Kevlar–carbon fabric with a density of 252 g/m^2^ [71].

A twill fabric of type 3 × 1, made of carbon and Kevlar fibres, is shown in Figure 4, which provides a high coverage capacity in manufacturing process. This type of Kevlar–carbon hybrid fabric is utilized to improve the strength, stiffness and abrasion protection of impactor or impacted elements made of composite laminates, in applications such as canoes, kayaks, seats, panels and others.

### 4.3. Braided Hybrid Kevlar-Carbon Fabrics

**Figure 5:**
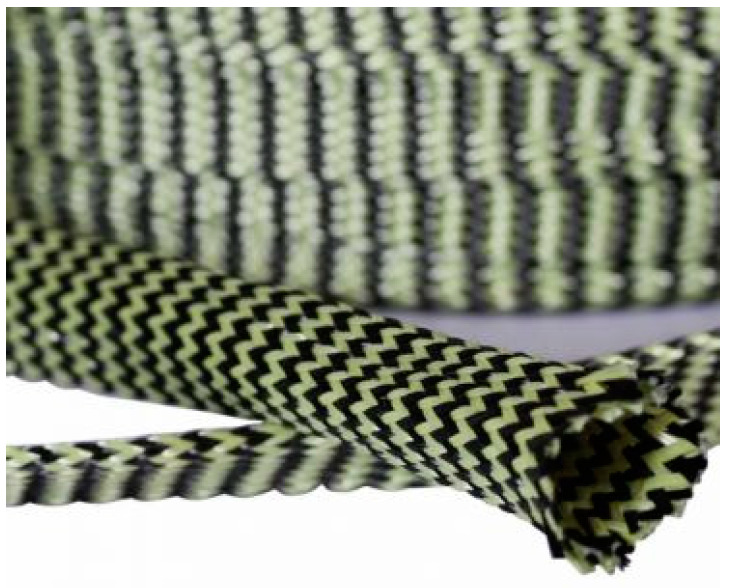
Braided hybrid Kevlar–carbon fabric [73].

The hybridization of the carbon and Kevlar fibres can be also achieved in the form of a ring-shaped braided cover (Figure 5).

## 5. Mechanical Characteristics of Kevlar–Carbon Hybrid Composite Materials

For applications requiring structures with a reduced thickness, low weight and high strength, the use of Kevlar–carbon hybrid composite materials is an ideal solution combining the advantages of individual fibre types. In addition, the simultaneous mitigation of their less desirable disadvantages is achieved using these two types of fibres as reinforcement materials [25,26,74,75].

The current research position shows that there is experimental research which has approached the mechanical testing of Kevlar–carbon composite materials for two cases of layer layup: the alternation of layers reinforced with a single type of reinforcing fibre; reinforcing all layers with a Kevlar–carbon hybrid fabric. In Table 11 [76], the mechanical characteristics for such Kevlar–carbon hybrid composite materials are illustrated.

It can be seen that the flexural strength is the highest for the composite material reinforced with hybrid Kevlar–carbon woven fabric (8CK twill), approximately 27% higher than the results obtained for the multi-layered hybrid composite materials (2K/8C/2K). In the case of hybrid Kevlar–carbon composite materials reinforced with Kevlar–carbon hybrid woven fabric in the same layer, the high stiffness of the carbon fibres combined with the high tenacity of Kevlar fibres leads to composite materials with very good mechanical characteristics.

Over the last 20–30 years, the researchers have focused on studying the advantages and disadvantages of hybridization of the carbon fibres and Kevlar fibres, in particular regarding the performances of such hybrid composite materials in impact tests. Both the carbon and Kevlar fibres have demonstrated high tensile strength. Additionally, the carbon fibres provide high stiffness while the Kevlar fibres assure high impact strength. Most researchers have focused on the comparative study of the behaviour of such hybrid composite structures in impact tests by replacing layers reinforced with carbon fibres with layers reinforced with Kevlar fibres. Table 12 [76] summarizes some of the values of the strain energy absorbed in low velocity impact tests, both for multi-layered hybrid composite materials and for composites reinforced either with carbon fibres or with Kevlar fibres. An instrumented falling weight impact equipment was used in the illustrated research [79].

The experimental results (Table 12) show that the hybridization of the carbon and Kevlar fibres in the composite structure leads to a value of 3.1 J for the absorbed strain energy, which is 29.17% or 10.71% higher than for the composites reinforced either with just carbon fibres or just with Kevlar fibres, respectively.

To observe the advantages and disadvantages of hybridization of the carbon fibres with those of Kevlar fibres in the layered composite structures, a series of impact tests were carried out on different types of sandwich composite structures whose core was made of paper honeycomb. For this purpose, a reference sandwich structure coded CF was chosen, with the lower and upper face sheets comprising four layers of composite material reinforced with plain carbon fabric. Then, one by one, the carbon-reinforced layers were replaced just for the upper shell face of the structure, with Kevlar49 fibres and with Kevlar–carbon hybrid fabric, in order to obtain the samples necessary for comparative impact tests, as shown in Table 13 [80].

The experimental data (Table 13) showed an improvement in the performance of the sandwich structure subjected to the impact tests after replacing some carbon-reinforced layers of the upper sheet face, either with Kevlar49 reinforced layers or with Kevlar–carbon hybrid fabric reinforced layers. At the same time, there was a decrease in the moduli of elasticity E1 and E2 in the directions corresponding to the two yarns of the reinforcement fabric (Table 13). It can also be seen that using either Kevlar fibres or hybrid Kevlar–carbon fabrics in the structure led to the values of the strain energies absorbed in impact test measuring up to 12.6% or 5.8% higher, respectively, when compared to the value of 31 J recorded for CF samples.

## 6. Structural Applications of Composite Materials Reinforced with Carbon and Kevlar Fibres in Civil Engineering

### 6.1. Current Trends in the Design of the Load-Bearing Structural Beams Strengthened with Composite Materials

Over the years, due to several advantages, composite materials have also begun have an important role in civil engineering, particularly for the consolidation of load-bearing structural elements made of different conventional materials (concrete, wood, bricks or steel). The most common composite structures which are easily used for consolidating and reinforcing both concrete and wood structural elements are pultruded fibre-reinforced polymer (FRP) composites. FRPs have become an attractive alternative especially for steel in civil engineering, owing to their high strength-to-weight ratio, stiffness-to-weight ratio and superior fatigue and corrosion resistance. The advantages of FRP materials are that they can be used either on the surface of the structural elements [81,82,83] or directly inserted into the structural elements [84]. The most common FRPs used in civil engineering are panels made of carbon fibre-reinforced polymer (CFRP). CFRP is a flat, hardened sheet, manufactured by pulling carbon fibres through epoxy, then, it is thermally treated in factory conditions. For concrete structural elements, CFRP bars has become a promising option for replacing steel reinforcement bars. In recent years, laminated structures reinforced with glass, Kevlar, basalt or hybrid fibres (glass–carbon, Kevlar–carbon) have also gained in popularity among civil engineers and have begun to be used as strengthening materials for structural elements [81,82,83].

**Figure 6:**
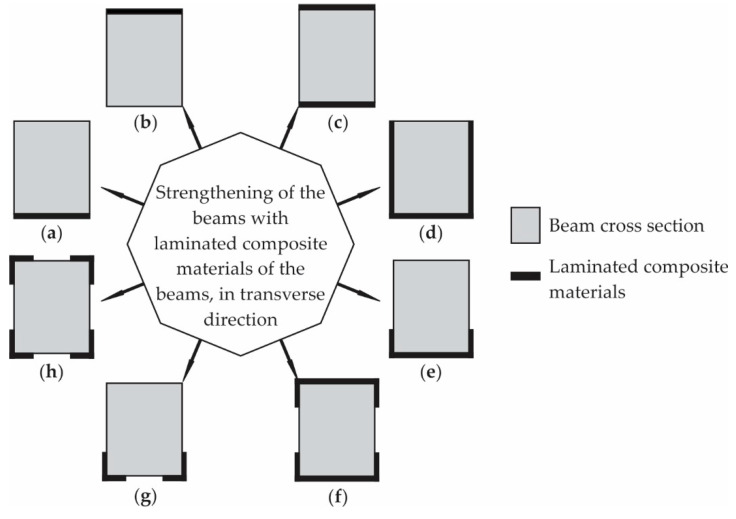
Section view of strengthening types of beams with laminated composite materials based on resins reinforced with fibres, located at different positions: (**a**) bottom; (**b**) top; (**c**) bottom and top sides; (**d**) bottom and lateral sides; (**e**) bottom side including bottom corners; (**f**) bottom and top sides including the corners; (**g**) bottom corners and (**h**) all corners.

As structural elements in a civil building, beams have the tendency to fail in two ways: flexural failure and shear failure. Figure 6 presents various methods of bonding different laminates (composite materials reinforced with fabrics or fibres) on the surface of the beam, in order to increase their capacity, depending on the specific requirements.

For example, in order to increase the impact properties of the beam, laminated composite materials can be applied, as described in Figure 6a–c. The shear strength capacity of concrete beams can be improved by externally applying laminated composite structures onto the beam surface, in the transversal direction to the concrete beam. To control the structural cracks, decrease the deflection and improve the fatigue strength, the laminated structures can also be applied in a longitudinal direction to the beam (Figure 6d,e). To obtain higher flexural rigidity, stiffness and increase the energy absorption capacity of the beams, laminate composite structures can be applied to the beam surface, as described in Figure 6f–h [81,82,83,85,86,87,88,89,90,91,92].

**Figure 7:**
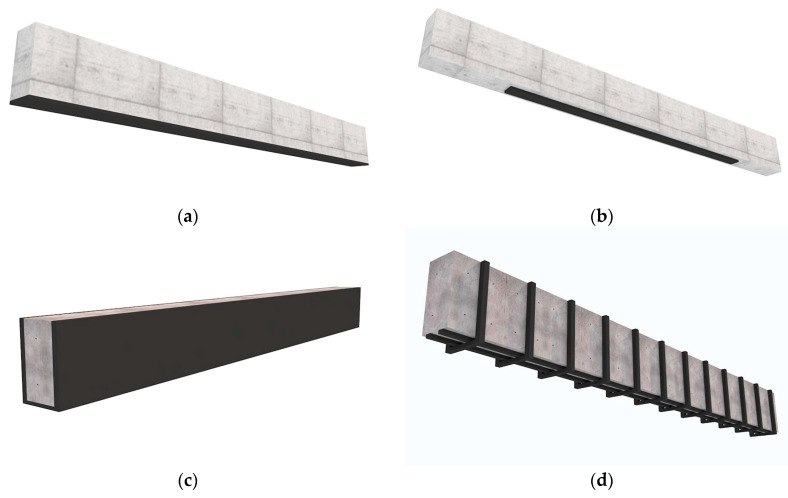
Lay-ups of the laminated composite materials on the longitudinal and transversal directions of the beam: (**a**) applied on the entire length of the beam; (**b**) partially and continuously applied on the beam length subjected to maximum bending moment; (**c**) applied on bottom and lateral beam faces like a U-shape and (**d**) partially and discontinuously applied on the beam length, at the position of the stirrups (just for concrete beams).

Figure 7 presents 3D sketch views of different types of strengthening the surface beams in longitudinal and transversal directions considering variations in length and width to meet the different needs of civil engineers. For example, bonding laminates on the bottom face continuously or partially (Figure 7a,b) increases the flexural strength of the beam. Additionally, bonding laminates on the bottom and lateral faces of the beam (Figure 7c) leads to an increase in both load-bearing capacity and flexural strength, while also limiting surface cracks on the beam. The bonding type shown in Figure 7d, is commonly used for consolidating concrete beams by applying laminates externally on the beam, in the same position as steel reinforcement (steel bars, callipers) was placed in the interior of the concrete beam, in order to increase the share strength and flexural strength capacity of that beam.

Bardak [84] demonstrated that strengthening an oak wooden beam by inserting layers of carbon fibres, glued with polyurethane adhesive, into the median longitudinal surface of the beam, leads to a decrease of approximately 50% in the deflection (vertical displacement) of the middle of the beam, compared with the unreinforced oak wooden beam. Although the rigidity of the reinforced wooden beam was improved, the carbon fibres do not have a significant effect on the strength and the delay of the initiation of the cracks because the normal stresses are equal to zero in the median surface. For this reason, it is recommended that the strengthening of wooden beams with carbon fibres is performed through gluing the fibres on the top and bottom faces of the beam.

### 6.2. Concrete Beams Strengthened with Carbon and Kevlar Composite Materials

Alagusundaramoorthy et al. [88] performed comparative four-point bending flexural tests on concrete beams strengthened at their bottom face with CFRP sheets or carbon fibre fabric to investigate the effectiveness of the externally bonded composite material concerning the increase in the flexural strength of the concrete beams. All concrete beams tested in that research [88] had cross-section dimensions of 230 × 380 mm^2^, a length of 4880 mm and were reinforced with different diameters of steel bars as follows: 25 mm diameter steel bars for tension in the longitudinal direction; 9 mm diameter steel bars for compression in the transversal direction. For strengthening the concrete beams, two different types of CFRP sheets were used: the first specimen of CFRP sheets were characterized by 1.40 mm thickness and 76 mm width and the second specimen of CFRP sheets were characterized by 4.78 mm thickness and 102 mm width. Both specimens of CFRP sheets had a 4270 mm length. The characteristics of the carbon fibre fabric used for strengthening the bottom of the concrete beams, in terms of dimensions, were 0.18 mm in thickness, 203 mm in width and 4370 mm in length. The bonding procedure was similar for both type of materials; firstly, the concrete surface was prepared via rubbing the beam surface with a sandpaper and then, all dirt and debris were removed with an air blower to obtain a clean surface. Secondly, the CFRP sheets and carbon fibre fabric were cleaned with acetone. Thirdly, an epoxy primer was applied on the concrete surface and left to dry for 30 min. Then, a two-component epoxy system was applied over this primer and also on the CFRP sheets and carbon fibre fabric. The CFRP sheets were glued onto the concrete surface and left to dry for 30 min. For the carbon fibre fabric, a supplementary thick layer of epoxy was applied over the fabric and after that, it was left to dry for 30 min. Before testing, all the concrete beams were cured for seven days at room temperature. A summary of the results from this research is presented in Table 14 [76].

It can be seen that there is an increase of up to 38.4% and 51.1% in the failure load in bending tests for beams strengthened with two or three CFRP sheets, respectively, bonded to the bottom surface of the concrete beams and subjected to tensile stresses. In comparison with the beam without any strengthening, for the concrete beams strengthened with carbon fibre fabrics, it can be observed there is an increase of up to 17.4% and up to 42.1% for one or two layers of carbon fabrics, respectively, applied on the bottom face of the beams. It was also shown that crack growth could be controlled.

Recent studies have focused on investigating the effects of Kevlar fibre-reinforced composites on the strengthening of the structural elements. However, these are not considered suitable for applications involving high compressive and bending stresses, as Kevlar fibres are known to have a tendency to bend and ultimately fail under such test conditions [12,93].

Recently, there has been growing interest among researchers in investigations on the method of strengthening concrete beams with Kevlar fibre laminates and their behaviour under bending and compression tests. In Table 15 [76], the experimental results obtained by Pandulu et al. [89] in flexural tests are summarized, in terms of the values of the breaking forces and in terms of the number of cracks developed in both the concrete beam and the beams strengthened with Kevlar layers. Comparative studies were conducted between concrete beams without strengthening and concrete beams that were strengthened on the bottom faces with Kevlar fabric. The dimensions of the cross section of the concrete beams were 200 mm in height and 150 mm in width, with a length of 1000 mm. The concrete beams were reinforced with 12 mm diameter steel bars in the longitudinal direction and with 8 mm diameter stirrups in the transversal direction. Kevlar fabric was used for strengthening the beams in one and two layers placed all over the bottom face and in a U shape (bottom and two lateral faces of the beam). Before bonding Kevlar fabric onto the concrete surface, the surface was cleaned with a wire brush to remove dust. Then, using an epoxy resin hardened with an agent, mixed in the ratio of 1:1, Kevlar fabric was laminated on the cured beam along the longitudinal reinforcement of the beam. Before testing, the concrete beams were cured for seven days at room temperature.

Considering the data from Table 15, an increase can be seen of up to 37.7% in terms of the failure load for the reinforced concrete beams strengthened with two layers of Kevlar laminates at the bottom of the beam and an increase of up to 88.4% for the concrete beams strengthened with two layers of Kevlar fabric in a U shape.

In another research study, Cakir et al. [90] showed that the load-bearing capacity of the concrete beams strengthened with hybrid Kevlar–carbon composite materials was approximately 4% higher than the one obtained for the same concrete beam without strengthening.

Tang et al. [91] analysed the impact performance of the concrete beams strengthened with carbon and Kevlar composite laminates at both top and bottom faces. In that research, to observe the role of each type of reinforcement material in the performance of the concrete beams, the impact tests were carried out on different types of beams: two reinforced concrete beams strengthened at the top and bottom faces with Kevlar fibre-reinforced composite material; two reinforced concrete beams strengthened at the top and bottom faces with carbon fibre-reinforced composite. For both of the beam configurations, repetitive impact tests were performed using a metal cylinder dropped from different heights onto the top surface of the beam. The results of the impact tests showed that the composite materials led to a significant increase in the impact strength capacity of the concrete beams. Additionally, the laminates reduced deformations and crack sizes. Comparing the test results obtained for beams strengthened with Kevlar fibres with the ones obtained for beams strengthened with carbon fibres, it was concluded that the impact strength depends on the type, thickness and properties of the composite materials used. In the case of those reinforced concrete beams, which were not strengthened with composite materials, the failure of the beam occurred after the appearance of cracks. In the case of those concrete beams reinforced with composite materials, even if cracks appeared in impact tests, the beams did not fail. This advantage is due to the use of Kevlar and carbon fibre laminates, which limit the opening of the cracks and also increase the shear strength.

Huang et al. [94] investigated the effects on the flexural behaviour of concrete beams of the following additional reinforcements to the internal steel rebar: (1) strengthening of the concrete beam with a U-shape profile made of hybrid laminated carbon/glass fibre-reinforced plastics (only the bottom face of the U shape is additionally reinforced with carbon fibres); (2) inserting a tube having a box cross section made of glass fibre-reinforced plastic (GFRP) inside the central part of the beam, subjected to compression stresses, covering approximately one third of the length of the beam. The main conclusion was that the U-shaped FRP mainly led to an increase in the load-bearing capacity (ultimate load) of up to 102.9% compared to the ordinary concrete beam containing just steel reinforcements, without any strengthening. It was reported that insertion of the GFRP tube in the concrete beam strengthened with a hybrid U-shaped profile improved the strength at the compressed part of the beam and therefore, the concrete did not crush at failure and such beams failed firstly by steel yielding, and secondly by CFRP breaking [94].

Regarding steel-reinforced concrete beams strengthened with Kevlar fibres, Mohana Sundari et al. [95] investigated the effects of wrapping in a U-shaped configuration containing one layer reinforced with one type of fibre (Kevlar, kenaf, basalt) on the flexural strength of such beams. It was shown that the flexural strength was higher by 23.5% for the beam strengthened with U-shaped wrapping with a Kevlar mat along its entire length compared to the strength obtained for the beam without strengthening. Moreover, it was shown that the flexural strength was greater by 5% for the beam strengthened with Kevlar fibres than for the beam strengthened with kenaf fibres and less by 6.6% than for the beam strengthened with basalt fibres [95].

### 6.3. Wooden Beams Strengthened with Carbon and Kevlar Composite Materials

Consolidation and rehabilitation of constructions containing wooden resistance elements have become a major concern for researchers worldwide. In recent years, more and more attempts have been made to use composite materials reinforced with carbon fibres to strengthen structural wood elements, to the detriment of steel due to their advantages including: low weight, anticorrosive properties, high resistance, easy manufacturing and easy assembly with other components.

Using the flexural tests with the four-points bending method, Li et al. [96] observed that bonding 1–3 layers reinforced with carbon fibres to the lower face of the wooden beam increased the bending strength by 39–61%, compared with the wooden beams which were not strengthened at the bottom face.

Similar to reinforced concrete beams, the strengthening of the bottom face of wooden beams may also be performed with sheets made of carbon fibre-reinforced plastics (CFRP).

Using bending tests, Torang and Desharma [92] investigated the influence of the bonding of the carbon fibre laminates to the lower surface of wooden beams on different lengths of the beam span. The test results summarizing the maximum force recorded at failure are presented in Table 16.

Analysing the results shown in Table 16, it can be seen that the maximum force value for the wooden beam with CFRP strengthening along its entire span is 48.3% higher than the value recorded for the regular wooden beam (without CFRP strengthening) having the same dimensions.

In a recently published paper [97], impact tests were carried out by drop test with a weight of 12 kg, from a height of 1.8 m, onto the top face of the wooden beams. Some wooden beams were strengthened at the bottom face, while others were not strengthened, according to the description shown in Table 17. During the impact tests, the beams were also subjected to an axial compressive force of 4 kN to improve their impact resistance. All wooden beams tested had the same length of 360 mm and the same rectangular cross section, having the dimensions of 36 mm × 45 mm.

Considering the experimental results (Table 17) obtained from impact tests, it may observe that the strengthening of wooden beams with carbon fibre-reinforced composite materials has a major influence on the impact strength. The strengthening with one, two or with eleven CFRP layers of the bottom face of the wooden beams led to an impact energy absorbed with a 46.56%, 182.17% or 103% higher value, respectively, than the value obtained for the wooden beam without CFRP strengthening.

Although the scientific literature lacks articles that provide research related to the strengthening of wooden beams with Kevlar fibres, Ou et al. [53] reported a good compatibility between grafted Kevlar29 fibres and wood flour (with grain of 425 μm) in the composite materials based on HDPE. It was shown that replacing with grafted Kevlar29 fibres of 3 wt.% of the total of 60 wt.% of reinforcement fibre content led to an increase up to 60.4% and 76.5% of the flexural strength and flexural modulus, respectively, compared to the composite reinforced just with wood flour (60 wt.%) [53]. The impact strength also increased up to 52.3% for the wood composite materials containing 3 wt.% ungrafted Kevlar29 fibres and 57 wt.% wood flour, compared to the impact strength obtained for wood composites unreinforced with Kevlar fibres [53].

## 7. Conclusions and Outlook

This paper is a review of the principal mechanical properties of carbon and Kevlar fibres and their composite materials, considering the results reported by different researchers in the literature. The aim of this paper is to highlight the contribution (advantages and disadvantages) of each type of reinforcement fibre, and to characterize the effects of the hybridization of these two types of fibres, carbon and Kevlar fibres.

The state of the art presented in this paper leads to some main conclusions:

- Kevlar–carbon hybrid composite materials combine the advantages of the individual components and, as a result, the resultant composite materials are characterized by high strength, high stiffness, good flexibility, high impact strength and a low coefficient of thermal expansion.
- Engineers have to take into account the environmental conditions when designing structural elements strengthened with Kevlar fibres because these are sensitive to the effects of the thermal changes and water absorption. It was shown that the tensile strength and Young’s modulus of Kevlar fibres at saturation with water can be usually up to 37% and 48% lower, respectively, than the values corresponding to dry fibres. On the other hand, Kevlar29 is less sensitive to water absorption.
- Reinforcing external layers of composite materials with Kevlar fabric or Kevlar–carbon fabric, which are hit by the impactor in impact tests, has led to an increase in absorbed strain energy.
- The results of the low velocity impact tests performed using composite materials reinforced with carbon and/or with Kevlar fibres have shown that for multilayer hybridization (carbon-reinforced layers alternating with Kevlar-reinforced layers), the absorbed strain energy can be 29.17% or 10.71% higher than the values recorded for composites reinforced either with only carbon fibres or with Kevlar fibres, respectively.
- Composite materials reinforced with carbon fibres, Kevlar fibres or hybrid Kevlar–carbon fabrics are increasingly used to strengthen structural elements and these have begun to play an important role in the structural behaviour of concrete and wooden beams.
- Increasing the number of layers of composite materials used to strengthen concrete beams has a positive influence on the load-bearing capacity of the beam, on the impact strain energy absorbed and last but not least, on modes of failure because the number of cracks decrease.
- Strengthening of wooden beams by bonding CFRP to the lower surface of the beam led to an increase in maximum flexural force of up to 48.3% with respect to the beam without strengthening. Absorbed strain energy also increased up to 182.2% due to the strengthening of the wooden beam with two CFRP layers on the lower surface of the beam.

The experimental results synthetically presented and comparatively analysed in this review article represent a source of documentation for civil engineers who design and build civil construction structures made of both concrete and wood. The data presented in this review paper provide the scientific background for the researchers interested in using carbon and Kevlar fibres and their composites to improve the load-bearing capacity of concrete or wooden beams. Civil engineers can also use the elastic and mechanical properties of materials, as presented in this article, for the numerical modelling of structures containing beams reinforced with such composite materials reinforced with carbon and Kevlar fibres.

The strengthening of the structural elements (wooden and concrete beams, panels) with composite materials reinforced with carbon and Kevlar fibres will become a common practice in the coming years due to both the versatility of the manufacturing technology, provided by the easy gluing of the structural elements on the surfaces, and due to the increase in the flexural strength and stiffness in bending loadings. Since the literature lacks publications on the mechanical properties of beams strengthened by gluing Kevlar–carbon hybrid fabric with polymeric adhesive, and subjected to bending loads or impact tests, this could be a good direction for further research starting from the current state analysis presented in this review article. The effects of water absorption on the mechanical behaviour of such structural elements could be also investigated in the future.

Although Kevlar fibres ensure improvements in the impact strength through absorbing a large amount of strain energy, the analysis of the data revised in this paper proves the degradation of their mechanical properties caused by moisture absorption. As a result, in further research, a solution for protecting the fibres against moisture absorption must be identified.

## Figures and Tables

**Table 1:** Tensile properties for different Kevlar fibres.

Fibre Name	Young’s Modulus *E* (GPa)	Poisson’s Coefficient *ν*	Tensile Strength *σ_t_* (GPa)	Extension to Break ε (%)	Density ρ (g/cm^3^)	References
Kevlar29	70	0.37	2.9	4.0	1.44	[42,43,44]
Kevlar49	135	0.36	2.9	2.8	1.45	
Kevlar100	60	-	2.9	3.9	1.44	
Kevlar119	55	-	3.1	4.4	1.44	
Kevlar129	99	-	3.4	3.3	1.45	
Kevlar149	143	0.30	2.3	1.5	1.47	
KevlarKM2	85	0.24	3.8	4.5	1.44	

**Table 2:** Characteristics of thermal degradation of Kevlar fibres.

Fibre Name	Heating Rate (°C/min)	Degradation Rate (%/min)		Temperature of Decomposition (°C)		Weight Reduction (Until Decomposition) (%)		Reference
		Air	Nitrogen	Air	Nitrogen	Air	Nitrogen	
Kevlar	variable	8.2	3.5	521.0	546.0	8	6	[45]
Kevlar29	20	26.0	21.0	449.1	480.0	9	8	[47]
Kevlar49	20	27.0	22.0	451.0	474.7	9	8	[47]
Kevlar49	10	-	-	482.0	538.0	9	8	[16]
Kevlar129	20	28.0	23.0	437.1	464.2	8	7	[47]

**Table 3:** Coefficient of thermal expansion for Kevlar fibres.

Fibre Name	Temperature Range (°C)	Coefficient of Thermal Expansion (×10^−6^ °C^−1^)		Reference
		Longitudinal	Transverse	
Kevlar29	25–150	−4.0	-	[16]
Kevlar49	25–150	−4.0	-	[16]
Kevlar49	20–80	−5.7	66.3	[48]

**Table 4:** The influence of water absorption on the mechanical properties of Kevlar fibres.

Fibre Name	Mechanical Properties for Dried Fibres		Mechanical Properties at Saturation		Water Absorption at Saturation	Reference
	Young’s Modulus *E* (GPa)	Tensile Strength *σ_t_* (GPa)	Young’s Modulus *E* (GPa)	Tensile Strength *σ_t_* (GPa)	(%)	
Kevlar29	70	2.9	64.0	2.48	5.3	[15]
Kevlar49	135	2.9	69.8	1.81	4.3	
Kevlar149	143	2.3	100.2	1.69	2.1	

**Table 5:** Effect of UV (ultraviolet) light on tensile strength of Kevlar fibres.

Fibre Size (Deniers) ^1^	UV Ray Exposure (h)	Tensile Strength Loss (%)	Reference
1500	450	65	[16]
3000		45	
4500		35	

^1^ Property unique to the fibre industries used to describe the fineness (and, conversely, the cross-sectional area) of individual filaments, thread, yarn, etc. It is defined as the weight expressed in grams, for 9000 m of a single filament.

**Table 6:** Properties of Kevlar short fibres.

Fibre Length (mm)	Colour	Density (g/cm^3^)	Work Temperature Range (°C)	Reference
0.5–1.0	yellow	1.45	−200/+350	[54]

**Table 7:** Example of a classification of carbon fibres.

Fibre Type	Main Characteristic	Young’s Modulus *E* (GPa)	Tensile Strength *σ*_max_ (GPa)	Reference
UHM	Ultra-high modulus	600–950	2.5–4	[32,33,34,56,57,58,59,60]
HM	High modulus	350–600	>2.5	
IM	Intermediate-modulus	280–350	>3.5	
HT	High tensile strength (or standard modulus)	200–280	>3.0	
LM	Low modulus	40–200	1–3.5	
SHT	Super-high tensile strength	-	>4.5	

**Table 8:** Influence of high temperatures on the tensile strength of carbon fibres.

Carbon Fibre Type	Temperature Exposure (°C)	Tensile Strength *σ_t_* (GPa)	Reference
UHM ^1^	room temperature	5.47	[63]
UHM ^1^	350	5.20	
UHM ^1^	450	4.27	
HT ^2^	room temperature	4.6	[65]
HT ^2^	2840	2.6	

^1^ UHM—Ultra-high modulus. ^2^ HT—High tensile strength.

**Table 9:** Coefficients of thermal expansion for carbon fibres.

Fibre Name	Precursor	Temperature Range (K)	Coefficient of Thermal Expansion (10^−6^/K)		Reference
			Longitudinal	Transverse	
P 100	Pitch	room temperature	−0.1	7.0	[66]
PANEX 33	PAN ^1^	350	1.0	5.0	
HTA 5131	PAN ^1^	450	0.8	6.0	
K-1100	Pitch ^1^	room temperature	−1.45	-	[32]
T 1000	PAN ^1^	room temperature	−0.55	-	
M40J	PAN ^1^	room temperature	−0.83	-	

^1^ PAN—Polyacrylonitrile.

**Table 10:** Electrical conductivity of PAN-based ^1^ carbon fibres.

Graphitization Temperature (°C)	Electrical Conductivity (S/cm)	Reference
1000	5.32	[68]
1800	51.01	
2200	75.91	

^1^ PAN—Polyacrylonitrile.

**Table 11:** Mechanical characteristics of hybrid composite materials reinforced with carbon and Kevlar fibres (adapted from [76]).

Composite Structure ^1^	Fibre Content	Matrix	Young’s Modulus *E* (GPa)	Tensile Strength *σ_t_* (GPa)	Flexural Strength *σ_b_* (GPa)	Poisson’s Coefficient *ν*_12_	Shearing Modulus of Elasticity *G*_12_ (GPa)	Reference
2K/8C/2K	60.0 vol.%	epoxy resin	-	1.25	1.150	-	-	[75]
3C/2K/2C/2K/3C	60.0 vol.%	epoxy resin	-	-	0.954	-	-	[75]
8CK plain	-	epoxy resin	11.34	-	-	0.090	1.49	[77]
8CK twill	45.0 wt.%	epoxy resin	35.25	4.07	4.180	0.141	-	[74]
CKCKC	37.2 vol.%	epoxy resin	-	2.00	0.430	-	-	[78]
KCKCK	20.2 vol.%	epoxy resin	-	2.40	0.500	-	-	[78]
KKCKK	38.4 vol.%	epoxy resin	-	2.60	0.600	-	-	[78]
CCKCC	42.0 vol.%	epoxy resin	-	2.70	0.460	-	-	[78]

^1^ C—carbon fibres; K—Kevlar fibres; CKCKC—hybrid composite material with 5 layers; 8CK plain—composite material reinforced with 8 layers of Kevlar–carbon plain hybrid fabric; 8CK twill—composite material reinforced with 8 layers of Kevlar–carbon twill hybrid fabric; 2K/8C/2K—composite hybrid material reinforced with 2 layers of Kevlar49 fibres, 8 layers of carbon HT fibres and 2 layers of Kevlar49 fibres; 3C/2K/2C/2K/3C—composite hybrid material reinforced with 3 layers of carbon HT fibres, 2 layers of Kevlar49 fibres, 2 layers of carbon HT fibres, 2 layers of Kevlar49 fibres and 3 layers of carbon HT.

**Table 12:** Absorbed strain energy in low velocity impact tests for composite materials reinforced with carbon fibres and/or with Kevlar fibres (adapted from [76]).

Composite Structure ^1^	Impact Speed (m/s)	Failure Mode	Absorbed Strain Energy at Impact ^2^ (J)	Reference
K-K	4.5	Penetrated	2.8	[79]
C-C		Penetrated	2.4	
C-K		Penetrated	3.1	
K-C		Penetrated	3.1	

^1^ #-#—two-layered composite material; C—carbon fabric; K—Kevlar fabric. All tested samples had the dimensions of 100 mm × 100 mm and were clamped with an annular support having the inner diameter of 75 mm; ^2^ values given are approximated from the graphs published in [79].

**Table 13:** Experimental results obtained for similar sandwich composite structures subjected to impact tests.

Code of Sandwich Structure	Description of the Upper Face Sheet of the Sandwich Composite	Young’s Modulus *E*_1_ (GPa)	Young’s Modulus *E*_2_ (GPa)	Absorbed Strain Energy at Impact (J)	Reference
CF	4 layers of composite material reinforced with plain carbon fabric	52.0	52.0	31.0	[80]
1K	1 layer of composite material reinforced with Kevlar49 fabric and 3 layers of composite material reinforced with plain carbon fabric	47.4	47.4	34.9	
2K	2 layers of composite material reinforced with Kevlar49 fabric and 2 layers of composite material reinforced with plain carbon fabric	42.5	42.5	33.2	
3K	3 layers of composite material reinforced with Kevlar49 fabric and 1 layer of composite material reinforced with plain carbon fabric	37.0	37.0	34.5	
4K	4 layers of composite material reinforced with Kevlar49 fabric	31.0	31.0	33.5	
1H	1 layer of composite material reinforced with hybrid Kevlar–carbon twill fabric and 3 layers of composite material reinforced with plain carbon fabric	50.25	45.5	32.4	
2H	2 layers of composite material reinforced with hybrid Kevlar–carbon twill fabric and 2 layers of composite material reinforced with plain carbon fabric	48.5	39.0	31.9	
3H	3 layers of composite material reinforced with hybrid Kevlar–carbon twill fabric and 1 layer of composite material reinforced with plain carbon fabric	46.75	32.5	33.1	
4H	4 layers of composite material reinforced with hybrid Kevlar–carbon twill fabric	45.0	26.0	32.8	

**Table 14:** Experimental results for concrete beams subjected to four-point bending flexural tests (adapted from [76]).

Beam Structure *	Size of CFRP Sheet/Carbon Fibre Fabric (Thickness and Width) mm × mm	Failure Load (kN)	Failure Modes	Reference
Concrete beam without CFRP/carbon fabric	-	190	Concrete crushing	[88]
Concrete beam with bottom surface strengthened with CFRP sheets (2 layers)	1.40 × 76	263	Concrete crushing and delamination at the CFRP-concrete interface	
Concrete beam with bottom surface strengthened with CFRP (3 layers)	1.40 × 76	287	Concrete crushing and delamination at the CFRP-concrete interface	
Concrete beam with bottom surface strengthened with carbon fabric (1 layer)	0.18 × 203	223	Carbon fabric breaking	
Concrete beam with bottom surface strengthened with carbon fabric (2 layers)	0.18 × 203	270	Carbon fabric breaking	

* The dimensions of the cross-section were 230 × 380 mm^2^ for all beams involved in that research.

**Table 15:** Experimental results for concrete beams subjected to flexural tests (adapted from [76]).

Beam Structure *	Failure Force (kN)	Number of Cracks	Failure Mode	Reference
Concrete beam without Kevlar layers	70.50	6	Flexural failure	[89]
Concrete beam with 1 Kevlar-laminated layer at bottom	84.06	3	Flexural failure and fabric rupture	
Concrete beam with 2 Kevlar-laminated layers at bottom	97.07	2	Flexural failure and fabric rupture	
Concrete beam with 1 Kevlar-laminated layer in U shape	119.26	No visible cracks	Flexural failure and fabric rupture	
Concrete beam with 2 Kevlar-laminated layers in a U shape	132.86	No visible cracks	Flexural failure and fabric rupture	

* The dimensions of the cross-section were 150 × 200 mm^2^ for all beams involved in that research.

**Table 16:** Experimental results on wood beams subjected to flexural tests.

Beam Structure	Beam Size *b* × *h* (mm × mm)	Maximum Force (N)	Reference
Wooden beam without CFRP strengthening	75 × 100	35,313.45	[92]
Wooden beam with CFRP strengthening along the entire span		52,378.00	
Wooden beam with CFRP strengthening along ¾ of the span		48,499.67	
Wooden beam with CFRP strengthening along ¼ of the span		36,864.77	

**Table 17:** Experimental results on wooden beams having the same dimensions, subjected to impact tests.

Beam Structure	Impact Strain Energy Absorbed (J)	Reference
Wooden beam without CFRP strengthening	48.67	[97]
Wooden beam strengthened with 1 layer of CFRP	71.33	
Wooden beam strengthened with 2 layers of CFRP	137.33	
Wooden beam strengthened with 11 layers of CFRP	98.80	

## Data Availability

Not applicable.

## References

[1] Dhas J.E.R., Arun M. (2022). A review on development of hybrid composites for aerospace applications. Mater. Today Proc..

[2] Reddy G.V., Naidu S.V., Rani T.S. (2008). Impact Properties of Kapok Based Unsaturated Polyester Hybrid Composites. J. Reinf. Plast. Compos..

[3] Thwe M.M., Liao K. (2002). Effects of environmental aging on the mechanical properties of bamboo-glass fiber reinforced polymer matrix hybrid composites. Compos. Part A-Appl. Sci. Manuf..

[4] Saba N., Tahir P.M., Jawaid M. (2014). A Review on Potentiality of Nano Filler/Natural Fiber Filled Polymer Hybrid Composites. Polymers.

[5] Zweben C. (1977). Tensile-strength of hybrid composites. J. Mater. Sci..

[6] Sathishkumar T.P., Naveen J., Satheeshkumar S. (2014). Hybrid fiber reinforced polymer composites—A review. J. Reinf. Plast. Compos..

[7] Ravishankar B., Nayak S.K., Kader M.A. (2019). Hybrid composites for automotive applications—A review. J. Reinf. Plast. Compos..

[8] Qu Z.T., Gao S.S., Zhang Y.J., Jia J.H. (2021). Analysis of the Mechanical and Preforming Behaviors of Carbon-Kevlar Hybrid Woven Reinforcement. Polymers.

[9] Nurazzi N.M., Asyraf M.R.M., Athiyah S.F., Shazleen S.S., Rafiqah S.A., Harussani M.M., Kamarudin S.H., Razman M.R., Rahmah M., Zainudin E.S. (2021). A Review on Mechanical Performance of Hybrid Natural Fiber Polymer Composites for Structural Applications. Polymers.

[10] Nunna S., Chandra P.R., Shrivastava S., Jalan A.K. (2012). A review on mechanical behavior of natural fiber based hybrid composites. J. Reinf. Plast. Compos..

[11] Priyanka P., Dixit A., Mali H.S. (2017). High-Strength Hybrid Textile Composites with Carbon, Kevlar, and E-Glass Fibers for Impact-Resistant Structures. A Review. Mech. Compos. Mater..

[12] Singh T.J., Samanta S. (2015). Characterization of Kevlar Fiber and Its Composites: A Review. Mater. Today-Proc..

[13] Priyanka P., Dixit A., Mali H.S. (2019). High strength Kevlar fiber reinforced advanced textile composites. Iran. Polym. J..

[14] Kumar S., Gupta D.S., Singh I., Sharma A. (2010). Behavior of Kevlar/Epoxy Composite Plates Under Ballistic Impact. J. Reinf. Plast. Compos..

[15] Minoshima K., Tsuru K., Komai K. (1998). Influence of vacuum and water on tensile fracture behaviour of aramid fibres. Damage and Fracture Mechanics: Computer Aided Assessment and Control.

[16] Kevlar Aramid Fiber Technical Guide. https://www.dupont.com/content/dam/dupont/amer/us/en/safety/public/documents/en/Kevlar_Technical_Guide_0319.pdf.

[17] Simil T.S., Midhun A.J., James V.J., Lakshmidasan S.K., Usha K.M., Rakesh S. Study on Mechanical Properties of Carbon-Carbon Composites Developed for Aerospace Applications. Proceedings of the National Conference on Carbon Materials (CCM)—Carbon Materials for Energy Harvesting, Environment, Nanoscience and Technology (Carbon Materials).

[18] Zhang Z.H., Yang W.M., Cheng L.S., Cao W.Y., Sain M.N., Tan J., Wang A., Jia H.B. (2020). Carbon Fibers with High Electrical Conductivity: Laser Irradiation of Mesophase Pitch Filaments Obtains High Graphitization Degree. ACS Sustain. Chem. Eng..

[19] Svitak M., Veith P., Barcik S. Research and application of carbon fibers for timbering wooden beam. Proceedings of the 4th International Science Conference Woodworking Techniques.

[20] Stanescu M.M., Bolcu D., Cluca I., Dinita A. (2014). Non Uniformity of Composite Materials Reinforced with Carbon and Carbon-Kevlar Fibers Fabric. Mater. Plast..

[21] Shokrieh M.M., Daneshvar A., Akbari S., Chitsazzadeh M. (2013). The use of carbon nanofibers for thermal residual stress reduction in carbon fiber/epoxy laminated composites. Carbon.

[22] Hadar A., Baciu F., Voicu A.D., Vlasceanu D., Tudose D.I., Adetu C. (2022). Mechanical Characteristics Evaluation of a Single Ply and Multi-Ply Carbon Fiber-Reinforced Plastic Subjected to Tensile and Bending Loads. Polymers.

[23] Cotet A., Bastiurea M., Andrei G., Cantaragiu A., Hadar A. (2019). Mechanical and Thermal Behavior of Carbon Nanotubes/Vinyl Ester Nanocomposites. Mater. Plast..

[24] Cotet A., Bastiurea M., Andrei G., Cantaragiu A., Hadar A. (2019). Dry Sliding Friction Analysis and Wear Behavior of Carbon Nanotubes/Vinylester Nanocomposites, Using Pin-on-Disc Test. Rev. Chim..

[25] Sanjay M.R., Arpitha G.R., Yogesha B. (2015). Study on Mechanical Properties of Natural—Glass Fibre Reinforced Polymer Hybrid Composites: A Review. Mater. Today-Proc..

[26] Xu D.H., Cerbu C., Wang H.W., Rosca I.C. (2019). Analysis of the hybrid composite materials reinforced with natural fibers considering digital image correlation (DIC) measurements. Mech. Mater..

[27] Pincheira G., Canales C., Medina C., Fernandez E., Flores P. (2018). Influence of aramid fibers on the mechanical behavior of a hybrid carbon-aramid-reinforced epoxy composite. Proc. Inst. Mech. Eng. Part L-J. Mater.-Des. Appl..

[28] Wagih A., Sebaey T.A., Yudhanto A., Lubineau G. (2020). Post-impact flexural behavior of carbon-aramid/epoxy hybrid composites. Compos. Struct..

[29] Priyanka P., Mali H.S., Dixit A. (2021). Dynamic mechanical behaviour of kevlar and carbon-kevlar hybrid fibre reinforced polymer composites. Proc. Inst. Mech. Eng. Part C-J. Mech. Eng. Sci..

[30] Ramana M.V., Ramprasad S. (2017). Experimental Investigation on Jute/Carbon Fibre reinforced Epoxy based Hybrid Composites. Mater. Today-Proc..

[31] Peebles L.H. (1988). Carbon fibres: Structure and mechanical properties. Int. Mater. Rev..

[32] Emmerich F.G. (2014). Young’s modulus, thermal conductivity, electrical resistivity and coefficient of thermal expansion of mesophase pitch-based carbon fibers. Carbon.

[33] Newcomb B.A. (2016). Processing, structure, and properties of carbon fibers. Compos. Part A Appl. Sci. Manuf..

[34] Goodhew P.J., Clarke A.J., Bailey J.E. (1975). A Review of the Fabrication and Properties of Carbon Fibres. Mater. Sci. Eng..

[35] Tanner D., Fitzgerald J.A., Phillips B.R. (1989). The Kevlar story—An advanced materials case-study. Angew. Chem.-Int. Ed. Engl..

[36] Vinay H.B., Govindaraju H.K., Prashanth B. (2014). A Review on Investigation on the Influence of Reinforcement on Mechanical Properties of Hybrid Composites. Int. J. Pure Appl. Sci. Technol..

[37] Alann A., Robert K. Strengthening of timber beams using FRP, with emphasis on compression strength: A state of the art review. Proceedings of the APFIS2009.

[38] Saad K., Lengyel A. (2022). Strengthening Timber Structural Members with CFRP and GFRP: A State-of-the-Art Review. Polymers.

[39] Naser M.Z., Hawileh R.A., Abdalla J.A. (2019). Fiber-reinforced polymer composites in strengthening reinforced concrete structures: A critical review. Eng. Struct..

[40] Yuhazri M.Y., Zulfikar A.J., Ginting A. Fiber Reinforced Polymer Composite as a Strengthening of Concrete Structures: A Review. Proceedings of the 2nd International Conference on Industrial Manufacturing Engineering.

[41] Pawlak A.M., Górny T., Dopierała Ł., Paczos P. (2022). The Use of CFRP for Structural Reinforcement—Literature Review. Metals.

[42] Jassal M., Ghosh S. (2002). Aramid fibres—An overview. Indian J. Fibre Text. Res..

[43] Manigandan S., Nandita P.G., Teja D.K., Gunasekar P., Nithya S., Devipriya J. (2019). Numerical investigation of low velocity out of plane impact behavior of Kevlar composites. Mater. Today Proc..

[44] Potluri R., Paul K.J., Babu B.M. (2018). Effect of Silicon Carbide Particles Embedmenton the properties of Kevlar Fiber ReinforcedPolymer Composites. Mater. Today-Proc..

[45] Li X.G., Huang M.R. (1999). Thermal degradation of Kevlar fiber by high-resolution thermogravimetry. J. Appl. Polym. Sci..

[46] Kunugi T., Watanabe H., Hashimoto M. (1979). Dynamic mechanical-properties of poly(P-PHENYLENETEREPHTHALAMIDE) fiber. J. Appl. Polym. Sci..

[47] Liu X.Y., Yu W.D. (2006). Evaluating the thermal stability of high performance fibers by TGA. J. Appl. Polym. Sci..

[48] Rojstaczer S., Cohn D., Marom G. (1985). Thermal-expansion of Kevlar fibres and composites. J. Mater. Sci. Lett..

[49] Dixit P., Ghosh A., Majumdar A. (2019). Hybrid approach for augmenting the impact resistance of p-aramid fabrics: Grafting of ZnO nanorods and impregnation of shear thickening fluid. J. Mater. Sci..

[50] Ertekin M. (2017). Fiber Technology for Fiber-Reinforced Composites.

[51] Lertwassana W., Parnklang T., Mora P., Jubsilp C., Rimdusit S. (2019). High performance aramid pulp/carbon fiber-reinforced polybenzoxazine composites as friction materials. Compos. Part B Eng..

[52] Haro E.E., Odeshi A.G., Szpunar J.A. (2018). The Effects of Micro- and Nano-Fillers’ Additions on the Dynamic Impact Response of Hybrid Composite Armors Made of HDPE Reinforced with Kevlar Short Fibers. Polym. Plast. Technol. Eng..

[53] Ou R.X., Zhao H., Sui S.J., Song Y.M., Wang Q.W. (2010). Reinforcing effects of Kevlar fiber on the mechanical properties of wood-flour/high-density-polyethylene composites. Compos. Part A Appl. Sci. Manuf..

[54] Dupont. www.dupont.com/Kevlar/en_US/products/aramid_pulp.html.

[55] Tye L. (2016). The tensile behavior of high-strength carbon fibers. Microsc. Microanal..

[56] Bhatt P., Goel A. (2017). Carbon Fibers: Production, Properties and Potential Use. Mater. Sci. Res. India.

[57] Frank E., Hermanutz F., Buchmeiser M.R. (2012). Carbon Fibers: Precursors, Manufacturing, and Properties. Macromol. Mater. Eng..

[58] Wielage B., Richter D., Mucha H., Lampke T. (2008). Influence of Fabric Parameters on Microstructure, Mechanical Properties and Failure Mechanisms in Carbon-Fibre Reinforced Composites. J. Mater. Sci. Technol..

[59] Huang X.S. (2009). Fabrication and Properties of Carbon Fibers. Materials.

[60] Alam P., Mamalis D., Robert C., Floreani C., Brádaigh C.M.O. (2019). The fatigue of carbon fibre reinforced plastics—A review. Compos. Part B Eng..

[61] Krucinska I., Stypka T. (1991). Direct measurement of the axial Poisson ratio of single carbon-fibers. Compos. Sci. Technol..

[62] Dieter L., Herwig P., Oskar P., Martin M., Manfred B., Christian R. Poisson Ratio Carbon Fibers at the Microscopic and the Nanoscopic Scale. Proceedings of the Carbon Conference.

[63] Chung D.D.L. (1994). Carbon Fiber Composites.

[64] Zollner M., Lieberwirth H., Kempkes P., Fendel A. (2019). Thermal resistance of carbon fibres/carbon fibre reinforced polymers under stationary atmospheric conditions and varying exposure times. Waste Manag..

[65] Xiao H., Lu Y., Zhao W., Qin X. (2014). The effect of heat treatment temperature and time on the microstructure and mechanical properties of PAN-based carbon fibers. J. Mater. Sci..

[66] Pradere C., Sauder C. (2008). Transverse and longitudinal coefficient of thermal expansion of carbon fibers at high temperatures (300-2500 K). Carbon.

[67] Pradere C., Batsale J.C., Goyheneche J.M., Pailler R., Delhaire S. (2009). Thermal properties of carbon fibers at very high temperature. Carbon.

[68] Gupta A., Dhakate S.R., Pal P., Dey A., Iyer P.K., Singh D.K. (2017). Effect of graphitization temperature on structure and electrical conductivity of poly-acrylonitrile based carbon fibers. Diam. Relat. Mater..

[69] Sun Z.F., Geng D.X., Meng F.X., Zhou L., Jiang R.G., Zhang D.Y. (2023). High performance drilling of T800 CFRP composites by combining ultrasonic vibration and optimized drill structure. Ultrasonics.

[70] Ying E.Z., Zhou Z.H., Geng D.X., Shao Z., Sun Z., Liu Y., Liu L., Jiang X., Zhang D. (2019). High efficiency ultrasonic assisted drilling of CFRP/Ti stacks under non-separation type and dry conditions. J. Zhejiang Univ. Sci. A (Appl. Phys. Eng.).

[71] Easycomposites. https://www.easycomposites.eu/188g-plain-weave-carbon-kevlar.

[72] Hp-Textiles. https://shop.hp-textiles.com/shop/en/165g-m-Hybrid-Fabric-Plain--Carbon-Kevlar-HP-P167AC-1653.html.

[73] Rockwest Composites. https://www.rockwestcomposites.com/br-ca-group.

[74] Cerbu C., Ursache S., Botis M.F., Hadar A. (2021). Simulation of the Hybrid Carbon-Aramid Composite Materials Based on Mechanical Characterization by Digital Image Correlation Method. Polymers.

[75] Dorey G., Sidey G.R., Hutchings J. (1978). Impact properties of carbon fibre/Kevlar 49 fibre hybrid composites. Composites.

[76] Ursache S., Cerbu C., Hadar A., Dumbrava F. Aspects regarding the mechanical characteristics of the carbon-Kevlar composite materials. Proceedings of the the 9th International Conference on Advanced Composite Materials Engineering—COMAT 2022.

[77] Haidzir H., Majid D.L., Rafie A.S.M., Harmin M.Y. (2013). Modal Properties of Hybrid Carbon/Kevlar Composite Thin Plate and Hollow Wing Model. Appl. Mech. Mater..

[78] Karthik K., Rajamani D., Manimaran A., Udayaprakash J. (2021). Evaluation of tensile properties on Glass/Carbon/Kevlar fiber reinforced hybrid composites. Mater. Today-Proc..

[79] Jang B.Z., Chen L.C., Wang C.Z., Lin H.T., Zee R.H. (1989). Impact resistance and energy-absorption mechanisms in hybrid composites. Compos. Sci. Technol..

[80] Gustin J., Joneson A., Mahinfalah M., Stone J. (2005). Low velocity impact of combination Kevlar/carbon fiber sandwich composites. Compos. Struct..

[81] Garcia P.D., Escamilla A.C., Garcia M.N.G. (2013). Bending reinforcement of timber beams with composite carbon fiber and basalt fiber materials. Composites.

[82] Shekarchi M., Oskouei A.V., Raftery G.M. (2020). Flexural behavior of timber beams strengthened with pultruded glass fiber reinforced polymer profiles. Compos. Struct..

[83] Chen D., Liao Y.D., Zhang Y. (2010). Experimental Study of RC Beams Strengthened with Basalt Fiber Sheets. Chin.-Ger. Jt. Symp. Hydraul. Ocean. Eng..

[84] Bardak T. (2019). Effects of Different Advanced Engineering Materials on Deformation Behaviour of Wood Structural Materials. Bioresources.

[85] Han Y., Liu H.B., Guo T. Analysis of Bending Stiffness of Reinforced Concrete Beams Strengthened with Carbon Fiber Sheet. Proceedings of the International Conference on Civil, Architectural and Hydraulic Engineering (ICCAHE 2012).

[86] Takeda K., Mitsui Y., Murakami K., Sakai H., Nakamura M. (1996). Flexural behaviour of reinforced concrete beams strengthened with carbon fibre sheets. Compos. Part A Appl. Sci. Manuf..

[87] Karthik K., Rajamani D., Raja T., Subramani K. (2021). Experimental investigation on the mechanical properties of Carbon/Kevlar fibre reinforced epoxy LY556 composites. Mater. Today Proc..

[88] Alagusundaramoorthy P., Harik I.E., Choo C.C. (2003). Flexural behavior of R/C beams strengthened with carbon fiber reinforced polymer sheets or fabric. J. Compos. Constr..

[89] Pandulu G., Jayaseelan R., Jeganathan S. (2020). Performance of RCC Beams Laminated with Kevlar Fabric. Jordan J. Civ. Eng..

[90] Cakir F., Acar V., Aydin M.R., Aksar B., Yildirim P. (2021). Strengthening of reinforced concrete beams without transverse reinforcement by using intraply hybrid composites. Case Stud. Constr. Mater..

[91] Tang T.P., Saadatmanesh H. (2003). Behavior of concrete beams strengthened with fiber-reinforced polymer laminates under impact loading. J. Compos. Constr..

[92] Sitorus T., Desharma S. Analysis and experiments of the effect of reinforcement of wood beam using carbon fiber reinforced polymer against bending strength. Proceedings of the 4th International Conference on Sustainable Civil Engineering Structures and Construction Materials (SCESCM).

[93] Zweben C. (1978). Flexural strength of aramid fiber composites. J. Compos. Mater..

[94] Huang L., Zhang C., Yan L.B., Kasal B. (2018). Flexural behavior of U-shape FRP profile-RC composite beams with inner GFRP tube confinement at concrete compression zone. Compos. Struct..

[95] Mohana Sundaria S., Vigneshkannanb S., Kannanc C., Rangarajd A., Amuthae M., Prakhashf N., Lingeshwarang N. (2023). Performance analysis and experimental investigation on mechanical properties of RCC beam with fibre wrapping. Mater. Today Proc..

[96] Li Y.F., Xie Y.M., Tsai M.J. (2009). Enhancement of the flexural performance of retrofitted wood beams using CFRP composite sheets. Constr. Build. Mater..

[97] Liu W., Yu Y., Zhang Z.X., Liu C.Y., Tong Y. (2021). Impact resistance of CFRP-reinforced wood beams under axial force using a digital image correlation method. Compos. Struct..

